# Diabetes status-related differences in risk factors and mediators of heart failure in the general population: results from the MORGAM/BiomarCaRE consortium

**DOI:** 10.1186/s12933-021-01378-4

**Published:** 2021-09-28

**Authors:** Matti A. Vuori, Jaakko Reinikainen, Stefan Söderberg, Ellinor Bergdahl, Pekka Jousilahti, Hugh Tunstall-Pedoe, Tanja Zeller, Dirk Westermann, Susana Sans, Allan Linneberg, Licia Iacoviello, Simona Costanzo, Veikko Salomaa, Stefan Blankenberg, Kari Kuulasmaa, Teemu J. Niiranen

**Affiliations:** 1 Division of Medicine, University of Turku and Turku University Hospital, Kiinanmyllynkatu 2, 20521 Turku, Finland; 2 Department of Public Health and Welfare, Finnish Institute for Health and Welfare (THL), Helsinki, Finland; 3 Department of Public Health and Clinical Medicine, Umeå University, Umeå, Sweden; 4 Cardiovascular Epidemiology Unit, Institute of Cardiovascular Research, University of Dundee, Dundee, UK; 5 University Heart Center Hamburg, Hamburg, Germany; 6 Catalan Department of Health, Barcelona, Spain; 7 Center for Clinical Research and Prevention, Bispebjerg and Frederiksberg Hospital, Copenhagen, Denmark; 8 Department of Clinical Medicine, Faculty of Health and Medical Sciences, University of Copenhagen, Copenhagen, Denmark; 9 Department of Epidemiology and Prevention, IRCCS Neuromed, Pozzilli, Italy; 10 Research Center in Epidemiology and Preventive Medicine, Department of Medicine and Surgery, University of Insubria, Varese, Italy

**Keywords:** Diabetes, Cardiovascular disease, Heart failure, Mediation, Hazard, Risk, Biomarker

## Abstract

**Background:**

The risk of heart failure among diabetic individuals is high, even under tight glycemic control. The correlates and mediators of heart failure risk in individuals with diabetes need more elucidation in large population-based cohorts with long follow-up times and a wide panel of biologically relevant biomarkers.

**Methods:**

In a population-based sample of 3834 diabetic and 90,177 non-diabetic individuals, proportional hazards models and mediation analysis were used to assess the relation of conventional heart failure risk factors and biomarkers with incident heart failure.

**Results:**

Over a median follow-up of 13.8 years, a total of 652 (17.0%) and 5524 (6.1%) cases of incident heart failure were observed in participants with and without diabetes, respectively. 51.4% were women and the mean age at baseline was 48.7 (standard deviation [SD] 12.5) years. The multivariable-adjusted hazard ratio (HR) for heart failure among diabetic individuals was 2.70 (95% confidence interval, 2.49–2.93) compared to non-diabetic participants. In the multivariable-adjusted Cox models, conventional cardiovascular disease risk factors, such as smoking (diabetes: HR 2.07 [1.59–2.69]; non-diabetes: HR 1.85 [1.68–2.02]), BMI (diabetes: HR 1.30 [1.18–1.42]; non-diabetes: HR 1.40 [1.35–1.47]), baseline myocardial infarction (diabetes: HR 2.06 [1.55–2.75]; non-diabetes: HR 2.86 [2.50–3.28]), and baseline atrial fibrillation (diabetes: HR 1.51 [0.82–2.80]; non-diabetes: HR 2.97 [2.21–4.00]) had the strongest associations with incident heart failure. In addition, biomarkers for cardiac strain (represented by nT-proBNP, diabetes: HR 1.26 [1.19–1.34]; non-diabetes: HR 1.43 [1.39–1.47]), myocardial injury (hs-TnI, diabetes: HR 1.10 [1.04–1.16]; non-diabetes: HR 1.13 [1.10–1.16]), and inflammation (hs-CRP, diabetes: HR 1.13 [1.03–1.24]; non-diabetes: HR 1.29 [1.25–1.34]) were also associated with incident heart failure. In general, all these associations were equally strong in non-diabetic and diabetic individuals. However, the strongest mediators of heart failure in diabetes were the direct effect of diabetes status itself (relative effect share 43.1% [33.9–52.3] and indirect effects (effect share 56.9% [47.7-66.1]) mediated by obesity (BMI, 13.2% [10.3–16.2]), cardiac strain/volume overload (nT-proBNP, 8.4% [-0.7–17.4]), and hyperglycemia (glucose, 12.0% [4.2–19.9]).

**Conclusions:**

The findings suggest that the main mediators of heart failure in diabetes are obesity, hyperglycemia, and cardiac strain/volume overload. Conventional cardiovascular risk factors are strongly related to incident heart failure, but these associations are not stronger in diabetic than in non-diabetic individuals. Active measurement of relevant biomarkers could potentially be used to improve prevention and prediction of heart failure in high-risk diabetic patients.

**Supplementary Information:**

The online version contains supplementary material available at 10.1186/s12933-021-01378-4.

## Background

Diabetes mellitus is an established heart failure (HF) risk factor. Already in 1974, the Framingham Heart Study investigators observed five- and two-fold increases in HF risk in diabetic women and men compared to their non-diabetic counterparts [1]. In addition, a recent systematic review by Aune et al. that covered over 21 million participants reported a doubled HF risk among diabetic patients [2]. In fact, 45% of patients hospitalized for HF have diabetes [3]. This association between diabetes and HF has been historically considered to be driven by increased coronary atherosclerosis in diabetic individuals. However, in contrast to this assumption, HF risk is also elevated among diabetic individuals without coronary heart disease [4] and in diabetic patients under rigorous glycaemic control [5]. These findings suggest that factors apart from coronary atherosclerosis may contribute to the increased HF risk in diabetes.

Potential non-atherosclerotic cardiometabolic causes for the increased HF risk in diabetes include subclinical inflammation, obesity, alterations in the lipid and energy metabolism, endothelial dysfunction and a cardiac muscle disease seen in diabetes, termed diabetic cardiomyopathy, that is unrelated to hypertension, coronary artery disease, or dyslipidemia [6, 7]. Reduced kidney function and related physiologic alterations may also contribute to the HF risk in patients with diabetes through intravascular volume overload, altered reabsorption or excretion of filtered glucose and/or sodium [8]. In addition, vitamin D levels are often low in individuals with obesity or insulin resistance and are also related to a wide range of cardiovascular and metabolic disorders as well, but ascertaining links to certain disease states, such as HF, have been difficult to make due to the relatively high prevalence of low vitamin D levels [9, 10].

The potential underlying and mediating factors of HF in diabetes are numerous and remain understudied in large well-phenotyped population cohorts with long enough follow-up times needed for HF to develop. We hypothesize that using survival modeling and mediation analyses will help in clarifying the differences between diabetic and non-diabetic individuals in developing HF. This information could help us understand the pathophysiology and the disproportionately increased risk of HF among individuals with diabetes. To reach this goal, both conventional HF risk factors and circulating biomarkers were measured in a large multinational pool of population-based cohorts of 94,011 individuals (55,271 with biomarkers available) with follow-up times up to 28 years totaling 1,326,515 person-years in the whole cohort.

## Methods

### Study cohorts

The MORGAM (MOnica Risk, Genetics, Archiving and Monograph) project is a multinational collaborative study with harmonized data from population-based cohort studies. The project aims at exploring the relationships between the development of cardiovascular diseases, their classic and genetic risk factors and biomarkers, originating from the WHO MONICA [Multinational MONItoring of trends and determinants in CArdiovascular disease] projects [11, 12]. Follow-up data for incident hospitalization for HF and its risk factors for up to 29 years (median 14.1 years) are available for 115,868 individuals from 20 cohorts from 6 countries: the FINRISK Study from Finland (baseline data collection carried out every 5 years between 1982–2007 with biomarker assessment in 1997), the Northern Sweden MONICA Study (1986–2009), the DAN-MONICA from Denmark (1982–1992), the Moli-sani Study from Italy (2005–2010), and the Scottish Heart Health Extended Cohort [SHHEC] from the UK (1984–1995).

**Fig. 1:**
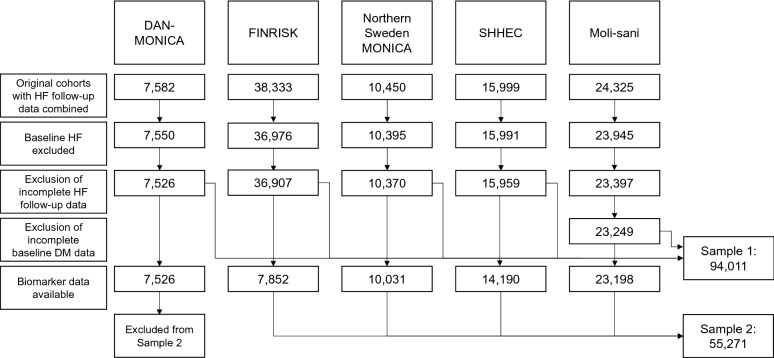
Flow chart for pooling population cohorts and exclusions made to form the population samples to be studied. Sample 2 is a subsample of Sample 1. All steps have been made for all cohorts but numbers after exclusions are illustrated only when the corresponding n is smaller due to them. *MONICA* Multinational Monitoring of trends and determinants in Cardiovascular disease, *SHHEC* Scottish Heart Health Extended Cohort, *HF* heart failure

After pooling these cohorts together, we derived two study samples that were included in the final analyses. Study sample 1 had complete HF follow-up data available (n = 94,011 after exclusions) and study sample 2 had also biomarker data available, being a subsample of the first one (n = 55,271 after exclusions; details in Fig. 1).

### Data collection

The diagnostic criteria for baseline disease states varies by cohort and baseline year but is mainly based on International Classification of Diseases (ICD) codes in hospital discharge data for all cohorts [13]. In general, baseline HF was defined with positive replies to survey questions (e.g., “Have you ever been diagnosed with…”, “Has a healthcare professional ever told you you have…”), hospital discharge register diagnoses (available in FINRISK, DAN-MONICA, SHHEC and Northern Sweden MONICA studies), or other health care information system (such as linkage with the nationwide Drug Reimbursement Register in FINRISK). Prevalent diabetes was based on self-report and/or register diagnoses (Additional file 1: Table S1a). Some early surveys did not differentiate between type 1 and type 2 diabetes and for this reason they were analyzed jointly (however, 16% of the diabetic participants were receiving insulin treatment at baseline). Detailed information on data collection, data harmonization, and definition of prevalent and incident disease is available for each cohort online [12].

### Biomarker data

Biomarkers used in this study were creatinine, C-reactive protein (hs-CRP), glucose, insulin, HDL cholesterol, LDL cholesterol, triglycerides, C-peptide, n-terminal atrial natriuretic peptide, type B (nT-proBNP), troponin I (hs-TnI), and vitamin D. High sensitivity assays were used for CRP and TnI. All biomarkers were sampled at baseline, deep-frozen and subsequently analyzed in the same central laboratory, except total cholesterol and HDL, which were mainly analyzed locally and during the same day as venous sampling (for Northern Sweden MONICA in 1999–2008, HDL cholesterol was measured from frozen samples in the aforementioned central laboratory). All lipids were serum measurements with > 10 h of fasting in Moli-sani and DAN-MONICA, > 4 h in FINRISK and Northern Sweden MONICA projects, while no fasting was required in SHHEC, which can mainly affect triglycerides levels. Low-density lipoprotein (LDL) cholesterol levels were calculated using the Friedewald formula without any additional hypertriglyceridemia-related adjustments. The laboratory methods and quality control results of the biomarkers have been published earlier [14].

### Follow-up

After excluding persons with HF at baseline from the study, the subjects were followed up for their first diagnosis of HF. The follow-up procedures relied on data from national population registers (except in Moli-sani), hospital discharge registers, causes-of-death registers, and death certificates. The follow-up periods started between 1982 and 2005 and ended between 2010 and 2015. Censoring was performed if the subject died from other cause or was lost to follow-up. Cohort-specific diagnostic criteria and follow-up period details with exact ICD codes used for HF and diabetes diagnosis at baseline and follow-up are combined in the Additional file 1: Table S1b and also provided online [12].

### Definitions

Smoking was defined as self-reported regular and occasional use of any of the following products: cigarettes, cigarillos, pipes, or cigars. Blood pressure (BP) was measured twice with a manual sphygmomanometer or an automated device and the mean of both measurements was used. Height and weight were measured with standard methods to calculate body mass index (BMI). Average alcohol use was calculated as grams per day (g/d) based on self-report.

### Statistical analyses

As the biomarker data were highly skewed to the right (i.e., a vast majority of cases were in the low or very low end of the range), we performed appropriate transformations (square root for lipid data and cubic root for the rest) and winsorizing (replacing the three highest values with the fourth highest) to avert further skewness caused by extreme outliers. After transformations, missing data were handled using multiple imputation with ten imputed data sets. We used random forests as the imputation method and ensured the convergence of the imputation algorithm and plausibility of imputed values by graphical inspection. After this, the continuous variables were centered by subtracting the variable mean from each variable and scaled by dividing the centered variable values by their standard deviations.

The unadjusted association between diabetes status and incident HF was assessed by estimating cumulative incidence curves for HF in the whole study sample 1 while the association between different risk factors and HF was examined using Cox proportional hazards models in study sample 2. The risk factors consisted of conventional HF risk factors (sex, alcohol use, systolic BP, BMI, smoking, baseline myocardial infarction [MI], and baseline atrial fibrillation [AF]) and several biomarkers (HDL cholesterol, LDL cholesterol, triglycerides, glucose, insulin, nT-proBNP, hs-TnI, creatinine, hs-CRP, and vitamin D). The analyses were performed separately for diabetic and non-diabetic individuals. We fitted models with different levels of adjustment to assess the impact of controlling for other variables. First, all risk factors were analyzed in separate models adjusted for conventional HF risk factors and stratified by cohort. Then, all risk factors were included as predictor variables in the same model and stratified by cohort. In addition, to test whether the association between the risk factor and HF was statistically different in diabetic versus non-diabetic individuals, an interaction term between a risk factor and diabetes status was included, in separate models for each risk factor. It is recommended to use age as time-scale instead of parametric adjustment as a covariate as parametric adjustment assumes that the connection between age and disease is known. In contrast, age as time-scale does not assume this and results in better visualization of the connection [15–17]. For this reason, age was used as the time-scale in all Cox models, resulting in age-adjustment.

**Fig. 2:**
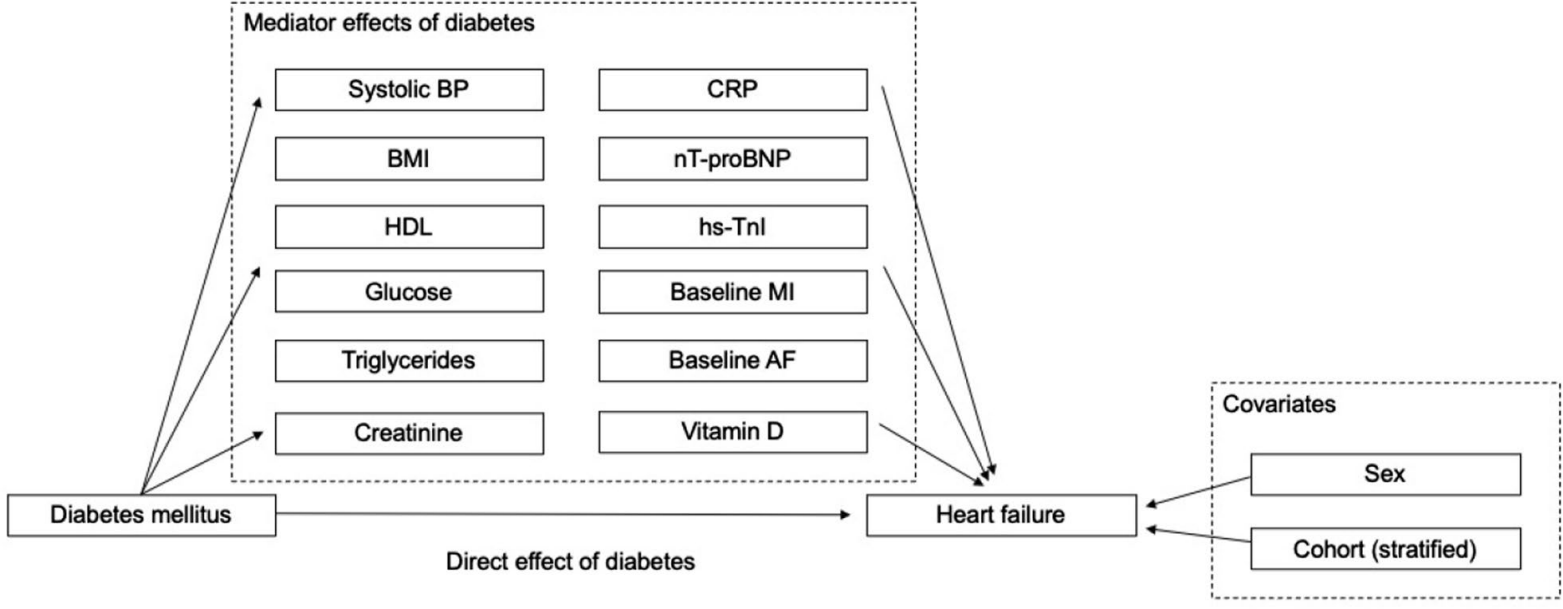
Conceptual model for mediation analysis. The effect of diabetes mellitus on the incidence of heart failure is divided to direct effect and mediator-driven effect. Covariates associated with the incidence of HF are depicted on the right. An individual’s age is taken into account by using age as the time-scale in the HF-free survival model. *HF* heart failure, *BP* blood pressure, *MI* myocardial infarction, *AF* atrial fibrillation, *CRP* C-reactive protein, *nT-proBNP* n-terminal atrial natriuretic peptide, type B, *hs* high-sensitivity assay, *TnI* troponin I

An exploratory mediation analysis with selected risk factors as potential mediator variables between diabetes and incident HF was performed in study sample 2. The conceptual model of the mediation analysis and the direct and indirect mediation effects are illustrated in Fig. 2. Baseline diabetes status (established before the baseline measurements) was used as the exposure variable and incident HF (during follow-up time) as the outcome variable, with the baseline biomarkers or conventional risk factors included as potential mediators of diabetes status on the outcome. We chose to exclude behavioral variables (smoking status and alcohol intake) from the mediation analysis as the effect of diabetes on HF onset cannot be expressed through them. To select the variables to be treated as potential mediators, we followed the approach presented by Yu et al. [18]. For a variable to be considered as a potential mediator, i.e., to be able to convey the predictor’s effect in the outcome, it needed to be significantly associated with both diabetes (exposure) and HF (outcome, with significance level of p < 0.1), when other variables were controlled for. When the aforementioned tests were not passed for a variable, it was used as a covariate by default. After applying these rules, systolic BP, BMI, HDL cholesterol, glucose, triglycerides, creatinine, CRP, nT-proBNP, hs-TnI, baseline MI, baseline AF, and vitamin D were included as mediator variables whereas sex and cohort were included as covariates.

All analyses were performed using R statistical software, version 3.5.1 [19]. The mediation analysis was performed using the mma-package [20] and multiple imputation using the mice-package [21].

## Results

**Fig. 3:**
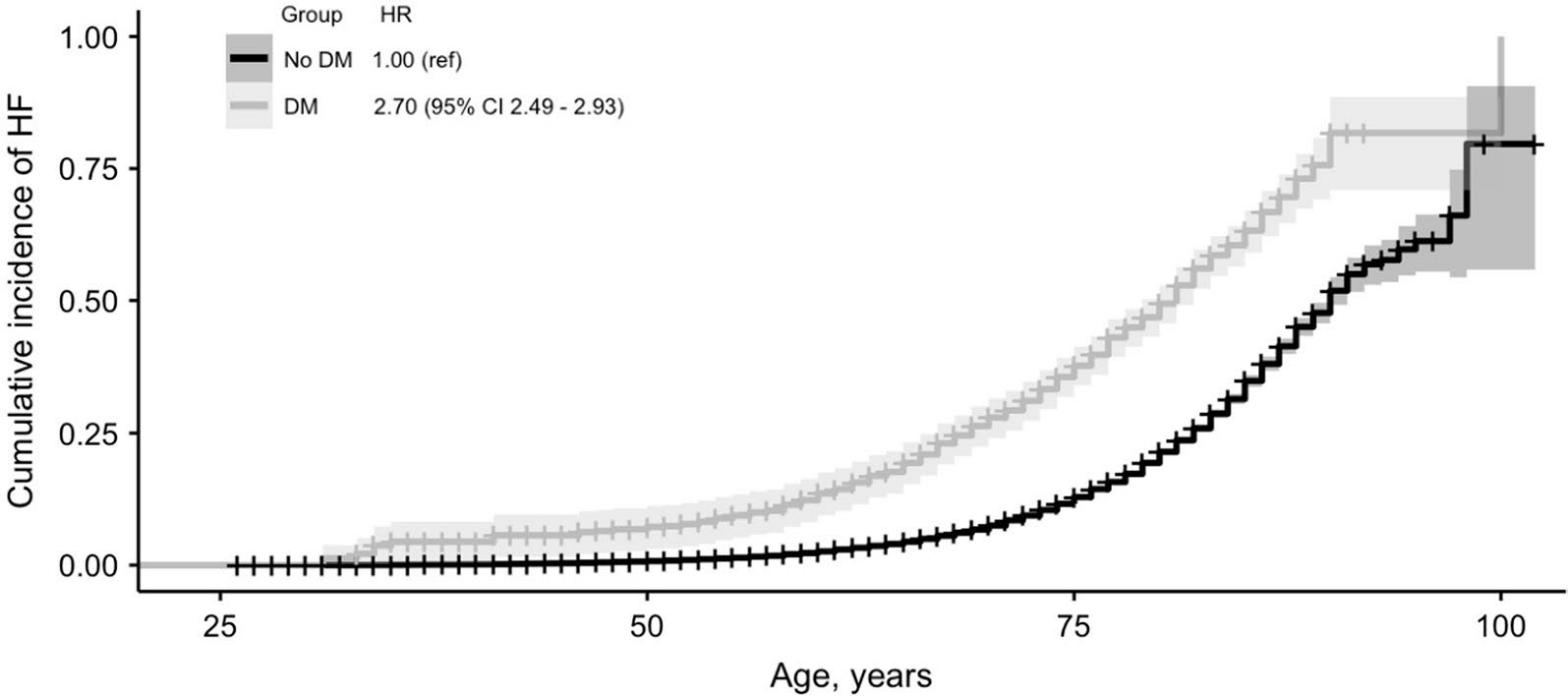
Unadjusted cumulative HF incidence curves for diabetic and non-diabetic individuals in the study sample 1. Total number of participants in analyses is 94,011. A total of 652 (17.0%) and 5524 (6.1%) cases of incident heart failure were observed in 3834 and 90,177 individuals with and without diabetes, respectively. Shaded area represents the 95% confidence interval and ticks the censored subjects. Log rank P value < 0.001. Numbers of events, censored events and individuals at risk at selected points are provided in Additional file 1: Table S2. *HF* heart failure, *HR* hazard ratio

The characteristics of the two study samples are presented in Table 1. In the full study sample (n = 94,011), 51.4% were women and mean age at baseline was 48.7 (standard deviation [SD] 12.5) years. Of this sample, 3834 (4.1%) individuals had baseline diabetes. Of persons with diabetes, 15.8% had insulin treatment, 37% had oral hypoglycemic medication, and 25.8% received only dietary treatment, suggesting that at least over 70% of these participants had type 2 diabetes. Incident HF was more common among diabetic individuals (n = 652; 17.0%) than among non-diabetic individuals (n = 5524; 6.1%) over a median follow-up time of 13.8 (interquartile range 5.7−22.8) years. Unadjusted cumulative HF incidence curves for diabetic and non-diabetic individuals in the study sample 1 over a follow-up time of up to 29.0 years are presented in Fig. 3. The hazard ratio (HR) for HF among diabetic individuals was 2.70 (95% confidence interval [CI], 2.49 − 2.93) when compared to non-diabetic participants.

**Table 1:** Characteristics of the study samples

	Sample 1 (whole sample: DAN-MONICA, FINRISK, Moli-sani, SHHEC, Northern Sweden MONICA)				Sample 2 (sample with biomarker data available: FINRISK, Moli-sani, SHHEC, Northern Sweden MONICA)			
	Overall	Diabetes	No diabetes	Missing, *n* (%)	Overall	Diabetes	No diabetes	Missing, *n* (%)
Demographics								
n	94,011 (100)	3,834 (100)	90,177 (100)		55,271 (100)	2,472 (100)	52,799 (100)	
Cohort								
DAN-MONICA	7,526 (8.0)	178 (4.6)	7,348 (8.1)					
FINRISK	36,907 (39.3)	1,526 (39.8)	35,381 (39.2)		7,852 (14.2)	397 (16.1)	7,455 (14.1)	
Moli-sani	23,249 (24.7)	1,468 (38.3)	21,781 (24.2)		23,198 (42.0)	1,465 (59.3)	21,733 (41.2)	
SHHEC	15,959 (17.0)	276 (7.2)	15,683 (17.4)		14,190 (25.7)	239 (9.7)	13,951 (26.4)	
Northern Sweden MONICA	10,370 (11.0)	386 (10.1)	9,984 (11.1)		10,031 (18.2)	371 (15.0)	9,660 (18.3)	
Women	48,320 (51.4)	1,771 (46.2)	46,549 (51.6)		28,307 (51.2)	1,066 (43.1)	27,241 (51.6)	
Age, years	48.65 (12.5)	57.65 (11.7)	48.27 (12.4)		51.08 (12.2)	60.45 (11.4)	50.64 (12.1)	
Medication								
Antihypertensive treatment	13,522 (14.4)	1,632 (42.6)	11,890 (13.1)	3,284 (3.5)	9,833 (17.8)	1,199 (48.5)	8,634 (16.4)	412 (0.7)
Lipid lowering treatment	3,099 (3.3)	579 (15.1)	2,520 (2.8)	30,281 (32.2)	2,465 (4.5)	479 (19.4)	1,986 (3.8)	15,704 (28.4)
Diabetes treatment								
Oral		1,427 (37.2)		810 (21.1)		1,148 (46.4)		518 (21.0)
Insulin		607 (15.8)		810 (21.1)		388 (15.7)		518 (21.0)
Diet		990 (25.8)		810 (21.1)		418 (16.9)		518 (21.0)
Risk factors								
Alcohol use, g/d	10.82 (18.0)	10.60 (18.7)	10.83 (18.0)	2,343 (2.5)	12.13 (19.4)	12.41 (20.0)	12.12 (19.4)	1,937 (3.5)
BMI, kg/m^2^	26.17 (4.6)	29.16 (5.5)	26.05 (4.5)	806 (0.9)	26.61 (4.7)	29.56 (5.5)	26.47 (4.6)	97 (0.2)
Smokers	29,914 (31.8)	862 (22.5)	29,052 (32.2)	161 (0.2)	16,140 (29.2)	475 (19.2)	15,665 (29.7)	42 (0.1)
Systolic BP, mmHg	135.23 (20.6)	145.80 (22.0)	134.79 (20.4)	761 (0.8)	135.66 (20.8)	147.42 (22.0)	135.11 (20.6)	15 (0.0)
Baseline MI	2,194 (2.3)	303 (7.9)	1,891 (2.1)	289 (0.3)	1,450 (2.6)	215 (8.7)	1,235 (2.3)	228 (0.4)
Baseline AF	560 (0.6)	51 (1.3)	501 (0.6)	6,328 (6.8)	423 (0.8)	51 (2.1)	372 (0.7)	6,371 (11.5)
Biomarkers								
HDL, mmol/l					1.4 (1.2 − 1.7)	1.3 (1.1 − 1.5)	1.4 (1.2 − 1.7)	2996 (5.4)
LDL, mmol/l					3.0 (2.4 − 3.8)	2.9 (2.3 − 3.5)	3.0 (2.4 − 3.8)	3019 (5.4)
Triglycerides, mmol/l					1.2 (0.9 − 1.7)	1.4 (1.0 − 1.9)	1.20 (0.9 − 1.6)	2956 (5.3)
Insulin, pmol/l					6.6 (4.5 − 10.0)	9.6 (6.2 − 15.4)	6.5 (4.4 − 9.8)	1894 (3.4)
Glucose, mmol/l					4.9 (4.5 − 5.5)	7.3 (5.8 − 9.5)	4.9 (4.5 − 5.4)	3,466 (6.2)
Creatinine, μmol/l					70.7 (61.9 − 79.6)	72.5 (63.6 − 85.7)	70.7 (61.9 − 79.6)	1120 (2.0)
hs-CRP, mg/l					1.4 (0.6 − 2.9)	2.0 (0.9 − 4.3)	1.3 (0.6 − 2.8)	1109 (2.0)
nT-proBNP, pg/ml					48.3 (25.5 − 90.2)	67.1 (32.5 − 143.3)	47.7 (25.3 − 88.1)	6678 (12.1)
hs-TnI, pg/ml					2.5 (1.4 − 4.3)	3.2 (2.0 − 5.5)	2.4 (1.4 − 4.2)	2643 (4.8)
Vitamin D, ng/ml					16.2 (11.5 − 22.4)	15.3 (11.0 − 20.7)	16.2 (11.5 − 22.4)	2543 (4.6)

Data presented as *n* (% of either overall, diabetic, or non-diabetic population) for categorical and as mean (standard deviation) for continuous variables regarding demographics, diabetes treatment and risk factors, and as median (Q1-Q3) before transformations (square root for HDL, LDL and triglycerides, cube root for others), winsorizing and imputations for biomarkers. Missing data information is presented as *n* (% of overall) missing for demographics, risk factors and biomarkers, *n* (% of individuals with diabetes) for diabetes treatment, and for biomarkers also as number imputed. Values for continuous variables are from regular ANOVA with equal variance assumption and for categorical variables from chi-squared tests with continuity correction. p values for all values < 0.001 except for Alcohol use (p = 0.45 in Sample 1 and = 0.484 in Sample 2)

*NA* not available, *MONICA* Multinational Monitoring of trends and determinants in Cardiovascular disease, *SHHEC* Scottish Heart Health Extended Cohort, *MI* myocardial infarction, *AF* atrial fibrillation, *CHD* coronary heart disease, *BP* blood pressure, *HF* heart failure, *CRP* C-reactive protein; *nT-proBNP* n-terminal atrial natriuretic peptide, type B, *hs* high sensitivity assay, *TnI* troponin I, *HDL* HDL cholesterol, *LDL* LDL cholesterol﻿﻿

**Fig. 4:**
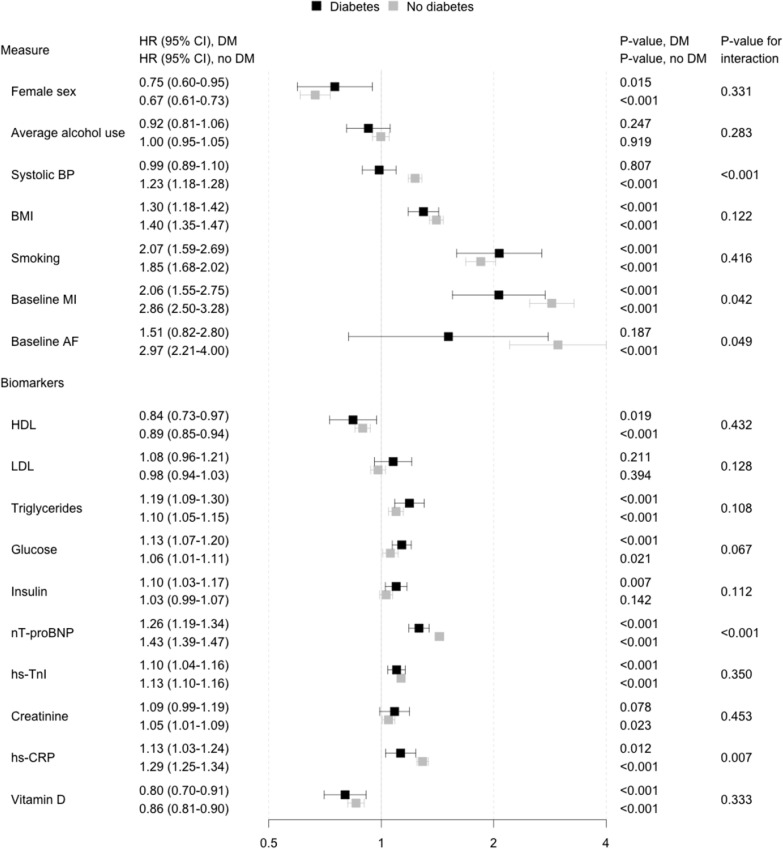
Association between risk factors and incident heart failure in diabetic and non-diabetic individuals with each risk factor analyzed in an independent model while adjusting for classical risk factors of heart failure. Models are adjusted for sex, alcohol consumption, systolic blood pressure, BMI, baseline myocardial infarction, baseline atrial fibrillation and stratified by cohort. Total number of participants in analyses is 55,271. A total of 319 (12.9%) and 2175 (4.1%) cases of incident heart failure were observed in 2472 and 52,799 individuals with and without diabetes, respectively. *HR* hazard ratio, *CI* confidence interval, *HF* heart failure, *BP* blood pressure, *MI* myocardial infarction, *CHD* coronary heart disease, *AF* atrial fibrillation, *CRP* C-reactive protein, *nT-proBNP* n-terminal atrial natriuretic peptide, type B, *hs* high sensitivity assay, *TnI* troponin I

**Fig. 5:**
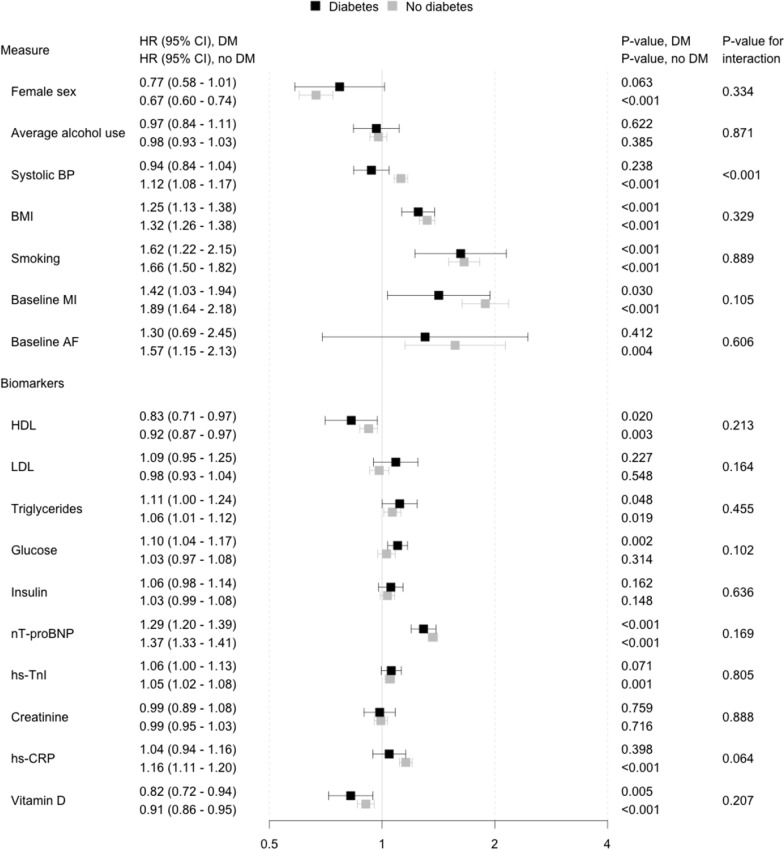
Association between risk factors and incident heart failure in diabetic and non-diabetic individuals with all risk factors included in the same model while adjusting for classical risk factors of heart failure. Total number of participants in analyses is 55,271. A total of 319 (12.9%) and 2175 (4.1%) cases of incident heart failure were observed in 2472 and 52,799 individuals with and without diabetes, respectively. *HR* hazard ratio, *CI* confidence interval, *HF* heart failure, *BP* blood pressure, *MI* myocardial infarction, *CHD* coronary heart disease, *AF* atrial fibrillation, *CRP* C-reactive protein, *nT-proBNP* n-terminal atrial natriuretic peptide, type B, *hs* high sensitivity assay, *TnI* troponin I

The associations of each risk factor with incident HF among diabetic and non-diabetic individuals are presented in Fig. 4 (separate models for each risk factor) and Fig. 5 (all risk factors included in the same model). In both analyses, we observed that conventional risk factors (male sex, BMI, smoking, prevalent MI, and prevalent AF) were more strongly related to HF in both diabetic and non-diabetic individuals than the circulating biomarkers, except for nT-proBNP which was associated with HF in both groups (HR 1.26 [CI 1.19 − 1.34] for diabetic and 1.43 [1.39 − 1.47] for non-diabetic individuals).

When the risk factors were analysed in separate models (Fig. 4), male sex, BMI, smoking, baseline MI, triglycerides, glucose, nT-proBNP, hs-TnI and hs-CRP were directly related to increased HF risk in both diabetic and non-diabetic individuals. HDL cholesterol and vitamin D were inversely related to the increased risk in both groups. Systolic BP, baseline AF and creatinine reached a statistically significant association with increased HF risk in non-diabetic individuals only whereas insulin levels were significantly associated with HF in diabetic individuals only. Interactions terms between diabetes status and systolic BP, baseline MI, baseline AF, nT-proBNP, and hs-CRP as HF risk factors were statistically significant and these associations tended to be stronger in the non-diabetic individuals. The p-value for the interaction term between glucose and diabetes status was 0.067 and thus trended towards significance. Alcohol use and LDL cholesterol were not related to HF risk in either diabetic or non-diabetic individuals in this study.

In the joint analysis with all risk factors included in the same model (Fig. 5), BMI, smoking, baseline history of MI, low HDL cholesterol, triglycerides, nt-proBNP, and low vitamin D were associated with increased HF risk in both diabetic and non-diabetic individuals. Systolic BP, baseline AF, hs-TnI, and hs-CRP were significantly linked to increased risk of HF in only non-diabetic individuals whereas glucose levels were significantly associated only in diabetic individuals. We observed a significant interaction between diabetes status and systolic BP for HF risk suggesting a stronger association in diabetic than in non-diabetic individuals. Alcohol use, LDL-cholesterol, insulin, and creatinine were not related to HF risk in either diabetic or non-diabetic individuals. A correlation matrix for all risk factors used in these analyses is reported in Additional file 1: Table S3. In sex-specific analyses, low alcohol intake was related to low risk of HF in women with diabetes (women: HR 0.64 [95 CI, 0.43–0.96]; men: HR 0.96 [95 CI, 0.80–1.14]). Heavy smoking was related to HF in both men and women with diabetes, but this association was particularly strong in women with diabetes (women: HR 3.96 [95 CI, 2.56–6.14]; men: HR 1.68 [95 CI, 1.21–2.34]). For biomarkers, the associations with HF were mainly similar in men and women, with the exception of nT-proBNP (women: HR 1.19 [95 CI, 1.08–1.31]; men: HR 1.31 [95 CI, 1.20–1.42]).

**Fig. 6:**
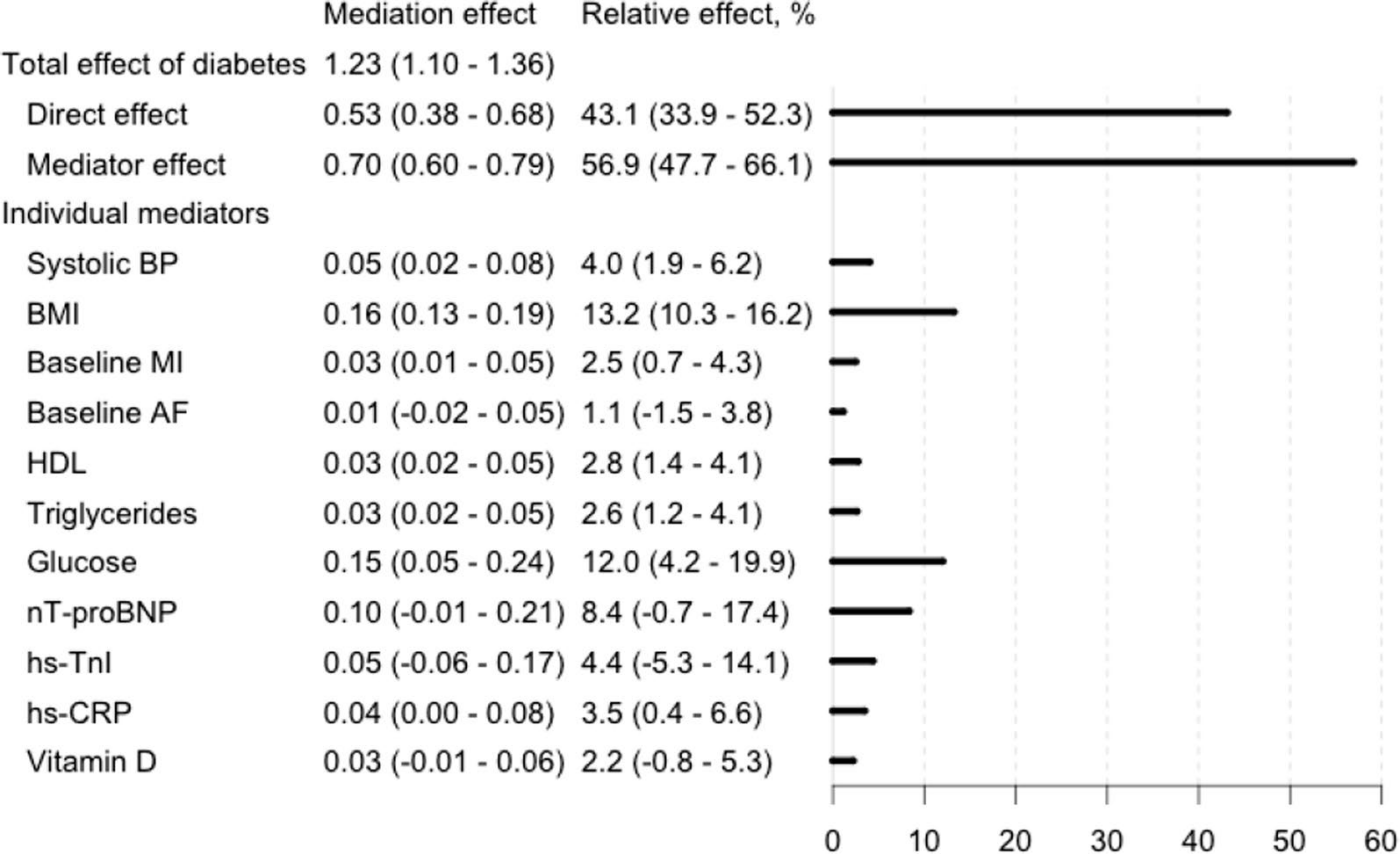
Direct and mediator-driven effects of diabetes mellitus on heart failure incidence. The 95% confidence intervals are reported in parentheses. The total effect of diabetes on heart failure risk (100%) is divided to the direct effect of diabetes and the combined mediator effect (see Fig. 2), with the individual mediators and their share of the mediation effect below. *HF* heart failure, *BP* blood pressure, *MI* myocardial infarction, *AF* atrial fibrillation, *CRP* C-reactive protein, *nT-proBNP* n-terminal atrial natriuretic peptide, type B, *hs* high-sensitivity assay, *TnI* troponin I

To further elucidate the effect of risk factors and the role of diabetes on HF incidence, we performed a mediation analysis with selected risk factors included as mediators of diabetes on the onset of HF in the sample with biomarker data (Fig. 6). The effect of diabetes status on HF risk was only partially explained by the mediators’ effects (relative effect of all mediators was 56.9% [95% CI 47.7 − 66.1%)] of the whole effect) and a considerable direct effect of diabetes (relative effect 43.1%; 95% CI 33.9 − 52.3%) was observed. The strongest mediator effects were seen with those represented by BMI (relative effect 13.2%; 95% CI, 10.3 − 16.2%), glucose (12.0%, 95% CI, 4.2 − 19.9%), and nT-proBNP (8.4%, 95% CI, -0.07–14.1%). Weak, but statistically significant mediation effects were also observed for the effects represented by systolic BP, HDL cholesterol, triglycerides, hs-TnI, hs-CRP, vitamin D and baseline histories of MI and AF. The effects of insulin and creatinine were selected as covariates by the algorithm (in addition to sex and stratified cohort, decided in advance).

## Discussion

In this study, our goal was to elucidate the key correlates and mediators of HF in diabetic individuals. This study confirms the strong association between diabetes and incident HF. Conventional cardiovascular disease risk factors, such as male sex, smoking, BMI, baseline MI, and baseline AF were the strongest correlates of incident HF in the Cox models. In addition, biomarkers for volume overload/cardiac strain (represented by nT-proBNP), myocardial injury (hs-TnI), and inflammation (hs-CRP) were also associated with incident HF. In general, all these associations were equally strong in non-diabetic and in diabetic individuals. However, the strongest indirect mediators of diabetes on incident HF were the effects represented by BMI, hyperglycemia, and volume overload/cardiac strain (the effect represented by nT-proBNP), and with a considerable direct effect of prevalent diabetes status itself as the biggest single driver of HF.

We expected to observe the strongest associations between traditional cardiovascular disease risk factors (such as sex, smoking, BMI, and prior cardiovascular disease) and incident HF in the Cox models. However, these factors were not in general more strongly related to HF in diabetic than in non-diabetic individuals nor were their effects strong mediators of diabetes on HF risk, with the notable exception for the effect represented by BMI. However, even though this relative risk per unit is similar, diabetic patients are likely to have higher BMI and BP levels, which lead to higher absolute risk of HF. Also, the majority of diabetic patients in our study received antihypertensive medication which may confound the association between BP and HF. Furthermore, smoking was one of the stronger risk factors for HF both in diabetic and non-diabetic individuals, especially in women. This observation is in accordance with a previous meta-analysis and it emphasizes the importance of smoking cessation in HF prevention [22]. The lack of association of HF with alcohol use is also in agreement with other studies [23]. Heavy drinkers, however, most likely do not take part in health studies adding a major confounding effect to studies researching associations of heavy drinking. These conventional cardiovascular disease risk factors do not, however, explain the greater than expected HF risk in diabetic patients [1].

The relation of hyperglycemia and HF among diabetic and non-diabetic populations has been assessed in various studies during the past two decades. The meta-analysis by Aune et al. concluded that the degree of hyperglycemia, even in prediabetic levels, is linearly associated with HF risk [2]. In addition, an improvement in glycemic control was shown to improve systolic and diastolic function in echocardiography in a study of 105 diabetes patients with poor glycemic control and no clinical HF [24]. Elevated levels of glucose have also been shown to increase the risk of diabetic cardiomyopathy [7]. In our study, increasing blood glucose levels were significantly associated with incident HF and this association trended towards being stronger in diabetic individuals (p = 0.067). Furthermore, the biggest mediation effect in our study was the direct effect seen with diabetes itself, and it together with the indirect mediation effect represented by glucose was driving 55.1% of the relative share of prevalent diabetes status’s effect on incident HF. There are several underlying mechanisms between hyperglycemia and HF risk. Foremost, hyperglycemia leads to increases in advanced glycated end-products in the heart muscle [7]. Hyperinsulinemia promotes cardiac hypertrophy even in healthy individuals and is a crucial part of the development of diabetic cardiomyopathy regardless of glucose levels [25]. Hyperinsulinemia also increases circulating triglycerides levels by activating lipolysis and by increasing the uptake of lipids in cardiomyocytes, resulting in lipotoxicity [26]. However, insulin did not pass mediation tests in our study – meaning it did not associate with both the predictor and outcome variables when other variables were controlled for. The physiological effect of insulin counteracts the deteriorating effect of hyperglycemia on cardiovascular outcomes which could explain why it failed the mediator tests. Furthermore, the heterogeneity of the diabetic population and insulin use might also be other reasons for our findings. The closer examination of the effects of hyperinsulinism on cardiac outcomes would require targeted analyses with type 2 diabetic individuals with endogenous hyperinsulinism. As glucose is also an osmotically active molecule, hyperglycemia leads to intravascular volume overload adding strain to the heart and activates the natriuretic peptide system. These mechanisms increase cardiac stress in a synergistic manner and are even more pronounced in obesity, which itself is also linked to hypervolemia [8].

The natriuretic peptide system acts in a wide spectrum of cardiovascular homeostatic mechanisms, and elevated levels are seen as a consequence in many diseases ranging from hypertension to renal failure [27]. Elevated levels of natriuretic peptides have been demonstrated to predict the onset of HF in asymptomatic individuals, diabetic and non-diabetic, in several studies [28–30]. These prior findings are supported by the indirect mediation effects seen with those represented by glucose and nT-proBNP in our study. Interestingly, natriuretic peptide levels correlate linearly with insulin sensitivity and are often diminished in diabetes and obesity, possibly due to faster clearance [31]. However, in addition to cardiac stretching/hypervolemia, natriuretic peptide levels are also known to increase in the same manner in these individuals in cardiac hypoxia, inflammation, and fibrotic remodeling, even when they are subclinical [27]. However, whether this finding reflects (1) the effects of obesity- and hyperglycemia-related hypervolemia in the circulatory system; (2) cardiospecific remodeling due to underlying cardiac disease associated with natriuretic peptide activity; or (3) the combination of these, cannot be distinguished in the setting of this study. Furthermore, HF with preserved (HFpEF) and reduced (HFrEF) ejection fractions are two different disease entities. HFpEF is more closely associated with diabetes and inflammation than HFrEF [32, 33]. In diabetes related HFpEF, diastolic dysfunction is often the first cardiac abnormality that can be observed [34]. However, echocardiography data were not available in this study.

HF pathogenesis in diabetes is driven to an extent by obesity, metabolic syndrome, and the related low-grade inflammation [35]. In this study, BMI was a major HF predictor and the indirect effect represented by it the most significant indirect mediator of the effect of diabetes on HF risk. In addition to BMI, the effect represented by hs-CRP was also a significant mediator of HF risk in diabetes. Obesity and subclinical inflammation have adverse effects on cardiac hemodynamics, structure, function and conduction that predispose to HF [36]. An extensive study of 83,021 type 2 diabetes patients without HF concluded that the risk of HF raises consistently and strongly with BMI and this elevation is 2–3 fold of what is observed in the non-diabetic population [37]. In the StrongHeart study, elevated CRP levels also predicted HF risk in individuals with diabetes or metabolic syndrome [38]. These studies highlight the importance of weight loss in the primary prevention of HF in overweight diabetic individuals. However, further research is needed to establish the links between obesity-related inflammatory biomarkers and HF in diabetes.

LDL cholesterol was not associated with HF, nor did it represent an indirect mediation effect of diabetes on HF in our study. However, as diabetic individuals are more likely to receive efficient cholesterol lowering treatment this might confound any potential links between cholesterol levels and HF risk. In a prior meta-analysis of 132,538 individuals in 17 trials concluded that LDL-lowering statin therapy had a protective role against new onset HF, regardless of whether a preceding MI had occurred [39]. In the Framingham Heart Study, increased HDL cholesterol concentration was associated with reduced HF risk and similar results were observed among diabetic participants of the Multiethnic Study of Aterosclerosis [40, 41]. As prior cardiovascular disease was strongly related to incident HF in our study, it appears that dyslipidemia-driven coronary atherosclerosis is still a major factor in the development of HF in diabetes, but not a major indirect mediator of diabetes’ effect on HF.

We observed a limited negative association and an indirect mediation effect between that represented by vitamin D and HF risk in our study. Vitamin D has a broad range of targets in the body and its deficiency has been described as associated with low-level inflammation, atherosclerosis and insulin resistance [10]. In a study of 12,215 participants of the Atherosclerosis Risk in Communities study, vitamin D deficiency was associated with two-fold HF risk in white, but not in black individuals [42]. However, in a recent meta-analysis on the effects of vitamin D on inflammatory markers in HF, Rodriguez et al. concluded that while vitamin D might have a role in the development of HF, evidence on the effect of vitamin D supplementation on clinical outcomes is lacking [43].

As the follow-up of our study extended across several decades, this study is the longest biomarker study in its size to address the relationship of diabetes and HF. However, it has some limitations that need to be considered. All the measurements were made at baseline, and we do not have information on risk factor and biomarker changes over time. Information on diabetes type at baseline and echocardiography data for assessing HF subtype and severity is also missing. In addition, as with all observational studies, a possibility of residual confounding remains due to variation in physical activity, diet, stress, and other factors that may not have been taken into account. Furthermore, our population-based pooled cohorts have substantial heterogeneity which on the other hand can also be considered as a strength of our study since our results can be regarded as pan-European and generalizable to the population of the continent at large. Nevertheless, the study population is dominantly white, and these results may not be generalizable to other ethnic groups. In addition, the exact diagnosis of HF varied by cohort. However, most of the studies relied on data derived from validated national healthcare registers [44–46].

## Conclusions

Our study adds weight to prior findings on diabetes being a strong predictor of HF in itself, and now backed also by a strong direct mediating effect demonstrated in our study. Conventional cardiovascular risk factors are strongly related to incident HF, but these associations are not in general stronger in diabetic compared to non-diabetic individuals. Our findings suggest that apart from the major direct mediating effect of diabetes, the main indirect mediators of HF risk conveyed by diabetes are the effects represented by obesity, hyperglycemia, and cardiac strain/volume overload. In light of our results and previous evidence, more aggressive weight management, glucose control, and cardiac screening are crucial in the primary prevention of HF in high-risk diabetic patients. There is unmet potential in the use of cardiac biomarkers for HF prediction in diabetes, too, but more evidence is needed on how these markers could be used more effectively in clinical decision-making.

## Supplementary Information

**Additional file 1: Table S1**. a Sources of HF and diabetes diagnoses. Diabetes includes all subtypes. b Detailed definitions for HF and diabetes diagnoses at baseline and for HF also during follow-up and the respective ICD codes used. Diabetes includes all subtypes. **Table S2.** Risk table for Figure 3. **Table S3.** Correlation matrix for all biomarkers and covariates used in analyses.

## Data Availability

The data are not available in a public repository. Access to the data is restricted by the ethical approvals and the legislation of the European Union and the countries of each study. Approval by the Principal Investigator of each cohort study and the MORGAM/ BiomarCaRE Steering Group will be required for release of the data. The MORGAM Manual gives more information on access to the data [47].

## Abbreviations

HF: Heart failure
DM: Diabetes mellitus
MORGAM: MOnica Risk, Genetics, Archiving and Monograph
WHO: World Health Organization
MONICA: Multinational MONItoring of trends and determinants in CArdiovascular disease
SHHEC: Scottish Heart Health Extended Cohort
ICD: International Classification of Diseases
CRP: C-reactive protein
HDL: High Density Lipoprotein
LDL: Low Density Lipoprotein
nT-proBNP: N-terminal atrial natriuretic peptide, type B
TnI: Troponin I
hs-: High sensitivity assay
BP: Blood pressure
BMI: Body mass index
MI: Myocardial infarction
AF: Atrial fibrillation
SD: Standard deviation
HR: Hazard ratio
CI: Confidence interval
NA: Not available
KELA: The Social Insurance Institution of Finland
