# Supplementary material for: Diabetes status-related differences in risk factors and mediators of heart failure in the general population: results from the MORGAM/BiomarCaRE consortium
Source: Cardiovasc Diabetol. 2021 Sep 28;20:195. doi: 10.1186/s12933-021-01378-4 (PMC8479921; doi:10.1186/s12933-021-01378-4)
Supplement: Supplementary file 1 — Additional file 1: Table S1. a Sources of HF and diabetes diagnoses. Diabetes includes all subtypes. b Detailed definitions for HF and diabetes diagnoses at baseline and for HF also during follow-up and the respective ICD codes used. Diabetes includes all subtypes. Table S2. Risk table for Figure 3. Table S3. Correlation matrix for all biomarkers and covariates used in analyses. [file 12933_2021_1378_MOESM1_ESM.docx]

**Additional file 1: Table S1a.** Sources of HF and diabetes diagnoses. Diabetes includes all subtypes.

| Cohort | DAN-MONICA | FINRISK | Moli-sani | Northern Sweden MONICA | SHHEC |
| --- | --- | --- | --- | --- | --- |
| At baseline (HF and diabetes): | | | | | |
| Hospital Discharge Register | X | X |  | X | X |
| National Diabetes Register |  |  |  | X (for diabetes diagnoses) |  |
| Drug Reimbursement Register |  | X |  |  |  |
| Self-reports | Only diabetes | HF and diabetes | HF and diabetes | Only diabetes | Only diabetes |
| During follow-up (HF): | | | | | |
| Hospital Discharge Register | X | X | X | X | X |
| Causes of Death Register | X | X | X | X | X |
| Follow-up period | 1982–2010 | 1982–2010 | 2005–2015 | 1986–2011 | 1984–2009 |
| Data source has been validated for HF diagnosis |  | X |  | X |  |

Abbreviations: NHS, National Health Service (UK); MONICA, Multinational MONItoring of trends and determinants in CArdiovascular disease; SHHEC, Scottish Heart Health Extended Cohort; KELA, The Social Insurance Institution of Finland.

**Additional file 1: Table S1b.** Detailed definitions for HF and diabetes diagnoses at baseline and for HF also during follow-up and the respective ICD codes used. Diabetes includes all subtypes.

| Cohort | DAN-MONICA | FINRISK | Moli-Sani | Northern Sweden MONICA | SHHEC |
| --- | --- | --- | --- | --- | --- |
| HF | | | | | |
| ICD-8 Codes | 427 428 | 427.00 * 427.10 * 428.99 * |  | 427.00 * |  |
| ICD-9 Codes |  | 402.9B * 414.8  428 | 428 | 428 | 428 |
| ICD-10 Codes | I11.0 I13.0 I13.2 I50 | I11.0 I13.0 I13.2 I50 |  | I11.0 I50 | I50 |
| Drug  reimbursement  codes |  | KELA code 201 ‡ |  |  |  |
| Diabetes | | | | | |
| ICD-8 Codes | 249 † 250 | 250 |  | National diabetes register used § |  |
| ICD-9 Codes |  | 250 | 250 |  | 250 |
| ICD-10 Codes | E10 E11 | E10 E11 E14 |  |  | E10 E11 E14 |
| Drug  reimbursement  codes |  | KELA code 103 ‡ |  |  |  |

Abbreviations: KELA, The Social Insurance Institution of Finland.

* Codes of the national modifications of ICD-8 and ICD-9 used in FINRISK/Northern Sweden MONICA:

427.00 = HF, cardiac oedema

427.10 = Acute pulmonary oedema
428.99 = Other forms of cardiomyopathies presenting with HF

402.9B = Hypertensive cardiomyopathy with HF

† In the Danish modification of ICD-8, there were two codes for insulin-dependent (249) and insulin-independent (250) diabetes mellitus from 1987 to 1993

‡ KELA Codes:
103 – Reimbursement for medical treatment in diabetes mellitus

201 – Reimbursement for medical treatment in chronic HF

§ For details on the Swedish National diabetes register:
Rolandsson O, Norberg M, Nyström L, Söderberg S, Svensson M, Lindahl B, et al. How to diagnose and classify diabetes in primary health care: Lessons learned from the Diabetes Register in Northern Sweden (DiabNorth). Scand J Prim Health Care. 2012 Jun 1;30(2):81–7.

**Additional file 1: Table S2.** Risk table for **Figure 3**.

| Diabetes status | Number of persons in the cohort | Age (years) | *N* at risk at the given age | % of the cohort at risk at the given age | Cumulative *n* of events by the given age | Cumulative *n* of censored subjects by the given age |
| --- | --- | --- | --- | --- | --- | --- |
| No diabetes | 90,177 | 30 | 6,461 | 7 | 0 | 150 |
|  |  | 50 | 31,977 | 35 | 203 | 17,309 |
|  |  | 70 | 23,925 | 27 | 2,422 | 61,309 |
|  |  | 90 | 212 | 8 | 5,506 | 84,530 |
| Diabetes | 3,834 | 30 | 78 | 2 | 0 | 0 |
|  |  | 50 | 623 | 16 | 18 | 265 |
|  |  | 70 | 1,149 | 30 | 319 | 1,910 |
|  |  | 90 | 8 | 0 | 651 | 3,177 |

**Additional file 1: Table S3.** Correlation matrix for all biomarkers and covariates used in analyses.

|  | Fe-male sex | Avg. alc. use | Sys. BP | BMI | Smo-king | Bl. MI | Bl. DM | Bl. AF | HDL | LDL | Trigl. | Gluc. | Ins. | nT-proBNP | hs-TnI | Creat. | hs-CRP | Vit. D |
| --- | --- | --- | --- | --- | --- | --- | --- | --- | --- | --- | --- | --- | --- | --- | --- | --- | --- | --- |
| Female sex | 1 | -0.39 | -0.14 | -0.08 | -0.08 | -0.09 | -0.04 | -0.02 | 0.3 | 0.01 | -0.17 | -0.18 | -0.08 | 0.25 | -0.26 | -0.49 | -0.01 | -0.11 |
| Avg. alcohol use | -0.39 | 1 | 0.1 | 0 | 0.11 | 0 | -0.01 | 0 | -0.01 | -0.03 | 0.08 | 0.1 | -0.01 | -0.09 | 0.09 | 0.21 | 0.02 | 0.07 |
| Systolic BP | -0.14 | 0.1 | 1 | 0.32 | -0.1 | 0.05 | 0.12 | 0.03 | -0.05 | 0.13 | 0.22 | 0.25 | 0.2 | 0.19 | 0.31 | 0.15 | 0.22 | -0.04 |
| BMI | -0.08 | 0 | 0.32 | 1 | -0.12 | 0.06 | 0.12 | 0.03 | -0.25 | 0.13 | 0.27 | 0.22 | 0.44 | 0 | 0.18 | 0.07 | 0.38 | -0.06 |
| Smoking | -0.08 | 0.11 | -0.1 | -0.12 | 1 | -0.01 | -0.05 | -0.03 | -0.08 | -0.03 | 0.1 | -0.05 | -0.06 | -0.05 | 0.01 | 0.01 | 0.05 | -0.1 |
| Baseline MI | -0.09 | 0 | 0.05 | 0.06 | -0.01 | 1 | 0.08 | 0.05 | -0.08 | -0.04 | 0.05 | 0.04 | 0.05 | 0.15 | 0.14 | 0.08 | 0.06 | 0 |
| Baseline DM | -0.04 | -0.01 | 0.12 | 0.12 | -0.05 | 0.08 | 1 | 0.03 | -0.07 | -0.03 | 0.07 | 0.25 | 0.12 | 0.07 | 0.07 | 0.04 | 0.07 | -0.03 |
| Baseline AF | -0.02 | 0 | 0.03 | 0.03 | -0.03 | 0.05 | 0.03 | 1 | -0.03 | 0 | 0 | 0.03 | 0.02 | 0.09 | 0.04 | 0.02 | 0.03 | 0.01 |
| HDL | 0.3 | -0.01 | -0.05 | -0.25 | -0.08 | -0.08 | -0.07 | -0.03 | 1 | 0.27 | -0.16 | -0.04 | -0.22 | 0.13 | -0.06 | -0.08 | -0.14 | 0.02 |
| LDL | 0.01 | -0.03 | 0.13 | 0.13 | -0.03 | -0.04 | -0.03 | 0 | 0.27 | 1 | 0.32 | 0.14 | 0.09 | -0.02 | 0.05 | 0.01 | 0.09 | 0.06 |
| Triglycerides | -0.17 | 0.08 | 0.22 | 0.27 | 0.1 | 0.05 | 0.07 | 0 | -0.16 | 0.32 | 1 | 0.22 | 0.31 | 0 | 0.25 | 0.25 | 0.24 | -0.05 |
| Glucose | -0.18 | 0.1 | 0.25 | 0.22 | -0.05 | 0.04 | 0.25 | 0.03 | -0.04 | 0.14 | 0.22 | 1 | 0.39 | 0.01 | 0.1 | 0.29 | 0.16 | 0.06 |
| Insulin | -0.08 | -0.01 | 0.2 | 0.44 | -0.06 | 0.05 | 0.12 | 0.02 | -0.22 | 0.09 | 0.31 | 0.39 | 1 | -0.07 | 0.07 | 0.12 | 0.27 | 0.03 |
| nT-proBNP | 0.25 | -0.09 | 0.19 | 0 | -0.05 | 0.15 | 0.07 | 0.09 | 0.13 | -0.02 | 0 | 0.01 | -0.07 | 1 | 0.19 | -0.06 | 0.14 | -0.03 |
| hs-TnI | -0.26 | 0.09 | 0.31 | 0.18 | 0.01 | 0.14 | 0.07 | 0.04 | -0.06 | 0.05 | 0.25 | 0.1 | 0.07 | 0.19 | 1 | 0.33 | 0.16 | -0.11 |
| Creatinine | -0.49 | 0.21 | 0.15 | 0.07 | 0.01 | 0.08 | 0.04 | 0.02 | -0.08 | 0.01 | 0.25 | 0.29 | 0.12 | -0.06 | 0.33 | 1 | 0.09 | 0.09 |
| hs-CRP | -0.01 | 0.02 | 0.22 | 0.38 | 0.05 | 0.06 | 0.07 | 0.03 | -0.14 | 0.09 | 0.24 | 0.16 | 0.27 | 0.14 | 0.16 | 0.09 | 1 | -0.04 |
| Vitamin D | -0.11 | 0.07 | -0.04 | -0.06 | -0.1 | 0 | -0.03 | 0.01 | 0.02 | 0.06 | -0.05 | 0.06 | 0.03 | -0.03 | -0.11 | 0.09 | -0.04 | 1 |

Data are presented as continuous variables except for categorical variables: sex (0=male, 1=female), smoking, and baseline diseases (0=no, 1=yes).

Abbreviations: Avg, average; Alc, alcohol; Sys, systolic; BP, blood pressure; BMI, body mass index; Bl, baseline; MI, myocardial infarction; AF, atrial fibrillation; Trigl, triglycerides; Gluc, glucose; Ins, insulin; nT-proBNP, n-terminal atrial natriuretic peptide, type B; TnI, troponin I; hs, high sensitivity assay; Creat, creatinine; CRP, C-reactive protein; Vit, vitamin.
